# Reframing Cervical Insufficiency as a Dynamic Process in the Preterm Birth Continuum: From Fixed Disease to a Modifiable Condition

**DOI:** 10.3390/diagnostics16020191

**Published:** 2026-01-07

**Authors:** Moon-Il Park

**Affiliations:** 1 Department of Obstetrics and Gynecology, Cervical Insufficiency Center, Dongtan Jeil Women’s Hospital, Hwaseong 18450, Republic of Korea; miparkmd@gmail.com; Tel.: +82-10-5225-5588; 2 Department of Obstetrics and Gynecology, Hanyang University Hospital, Seoul 04763, Republic of Korea

**Keywords:** cervical insufficiency, cervical remodeling, cervical biomechanics, cervical inflammation, diagnostic biomarkers, cerclage, preterm birth continuum

## Abstract

For decades, cervical insufficiency (CI) has been framed predominantly as a mechanical failure of the cervix resulting in painless mid-trimester dilatation. This disease-centered paradigm, reinforced by clinical teaching and administrative coding, does not fully capture the dynamic and biologically integrated nature of cervical remodeling. Accumulating evidence suggests that cervical change is governed by coordinated mechanical, inflammatory, and immunologic interactions rather than by a purely anatomic defect. To outline a process-oriented conceptual framework that situates CI within the broader preterm-birth continuum, this perspective aims to integrate biomechanical, inflammatory, and immunologic dimensions of cervical remodeling and to emphasize that infection- and inflammation-related changes represent dynamic, potentially modifiable elements that may inform more individualized, biology-guided clinical decision-making. This Perspective traces the evolution from a traditional “disease entity” interpretation of CI toward a more integrated view of cervical remodeling as a dynamic, biology-responsive process. Emerging data suggest that when intra-amniotic infection or inflammation is appropriately managed, cervical competence may be partially restored, and mechanical support can be applied more safely in selected patients. Clinical observations indicate that infection-controlled cerclage is associated with meaningful prolongation of gestation. Earlier reports describing double-level mechanical reinforcement techniques conceptually align with contemporary interpretations of infection-controlled emergent cerclage by linking surgical timing with the underlying biology of cervical change. Rather than proposing a prescriptive management pathway, this framework highlights how mechanical, inflammatory, and immunologic factors may interact across heterogeneous CI etiologies and how individualized intervention may be guided by biologic context. Understanding CI as a dynamic rather than a fixed condition provides a framework that integrates its mechanical, inflammatory, and immunologic dimensions within the preterm birth continuum. Such a perspective encourages individualized, biology-informed interpretation of cervical change and supports more context-specific use of established interventions such as cerclage. By emphasizing developmental processes rather than a static defect, this approach seeks to bridge classical clinical practice with contemporary insights into cervical remodeling.

## 1. Introduction

Historically, cervical insufficiency (CI) has been defined largely as a structural or mechanical failure of the cervix leading to painless mid-trimester dilation. While this framework provides a practical clinical anchor, it does not fully reflect evidence showing that cervical remodeling is shaped by interacting mechanical, inflammatory, immunologic, and microbiologic pathways. Foundational contributions by Romero et al. [1], Lee et al. [2], and Vink et al. [3] highlighted these biologic influences, but their work remained primarily descriptive rather than integrative, leaving the broader diagnostic paradigm essentially unchanged. As a Perspective, this article seeks not to propose a new disease construct but to outline a process-oriented conceptual framework in which CI is interpreted as a dynamic, biology-responsive condition rather than a fixed mechanical defect.

Clinical experience with emergency cervical cerclage (ECC) suggests that restoration of cervical competence depends not only on mechanical reinforcement but also on the biologic context in which the procedure is performed. When intra-amniotic infection or inflammation is appropriately addressed, cervical tissues may regain partial functional integrity, allowing mechanical support to be applied more safely in selected patients, as noted in observational cohorts [2]. This interpretation aligns with contemporary models describing cervical remodeling as a dynamic interplay of contractile, inflammatory, and extracellular matrix interactions [4,5,6,7]. From this perspective, the rationale for using two sutures in advanced ECC has been described not as a superiority claim but as a biologically informed attempt to stabilize the cervix by supporting both mechanical structure and residual mucosal elements. The potential for an intervening space between sutures to retain remnants of mucus or fibrin has been theorized to contribute to local biochemical and immunologic defense, although such mechanisms remain hypothetical and require further validation. In practical ECC settings, clinical care typically integrates antibiotic therapy with timely mechanical reinforcement in close succession, reflecting the need to address both biologic activation and impending structural failure.

Historically, anatomically defined true double-level cervical cerclage strategies were described in Europe in the late 1980s and were subsequently refined through clinical experience reported in East Asia around the late 1990s and early 2000s, particularly in cases of advanced cervical dilation or membrane prolapse [8,9]. These early reports described attempts to combine mechanical stabilization with preservation of residual mucosal elements, concepts that are broadly consistent with contemporary biologic interpretations of cervical remodeling. More recent comparative studies and systematic reviews [10,11,12,13] have examined two-suture approaches. Although the overall evidence base remains heterogeneous and largely observational, several analyses consistently report favorable trends in ECC, including greater gestational prolongation and lower preterm-birth rates in selected patients. These observations are also reflected in a recent systematic review by Park and Park [14], which noted that benefit signals for double level cerclage were most evident in ECC populations while remaining variable in prophylactic or less advanced presentations. Transabdominal cerclage may benefit selected subgroups, such as women with a prior failed transvaginal cerclage or anatomic constraints; however, existing evidence is limited by non-randomized designs and small sample sizes, preventing firm conclusions regarding its comparative effectiveness [15]. Collectively, these observations underscore the importance of interpreting cerclage outcomes within the broader biologic context of cervical remodeling rather than attributing benefit solely to technical variation.

Beyond procedural considerations, these observations invite a broader reconsideration of how CI is diagnosed and conceptualized. Increasing evidence indicates that CI does not arise from a single pathway but reflects heterogeneous biologic processes, including infectious or inflammatory activation, traumatic or postsurgical remodeling, postpartum healing disturbances, and genetic or connective-tissue variants. Because these etiologies follow distinct trajectories of cervical remodeling, their clinical manifestations and responses to intervention are unlikely to be uniform. Within this reframed perspective, diagnostic interpretation should move beyond purely structural findings to incorporate biologic indicators—such as inflammatory biomarkers, cervical elastography, and extracellular-matrix-related measures—that reflect active remodeling dynamics. If CI represents a biologically active condition rather than a static mechanical defect, then diagnostic assessment must capture the evolving interaction between cervical mechanics, host immune response, and tissue biology.

Within this context, the value of the present Perspective lies not in redefining established concepts, but in making explicit how diagnostic framing influences clinical interpretation, research design, and guideline translation. By situating cervical insufficiency within a dynamic diagnostic process rather than a static category, this framework seeks to highlight areas where existing evidence may be interpreted differently depending on underlying assumptions. Such clarification may be particularly relevant for readers navigating discrepancies between biologic insight, diagnostic practice, and guideline language.

## 2. Etiologic Pathways and the Biology of Cervical Remodeling

In earlier practice, intra-amniotic infection was widely regarded as a contraindication to cerclage, based on the assumption that mechanical closure in the presence of infection would precipitate preterm labor or amplify inflammation. This reflected the traditional view of CI as a static structural defect requiring mechanical correction. Educational and administrative conventions also reinforced this framework; CI continued to be defined as a discrete pathology within medical curricula and coding systems, which contributed to a binary clinical logic in which structural failure was emphasized over underlying biologic processes. Importantly, the considerations discussed here do not apply to cases of confirmed intra-amniotic infection or clinical chorioamnionitis, which remain indications for urgent delivery. Instead, this discussion pertains to stabilized or subclinical inflammatory states in which infection control has been achieved prior to considering mechanical reinforcement. Within these contexts, emerging data suggest that biologic modulation—particularly suppression of microbial and inflammatory activation—may allow safer application of cerclage in selected patients. Increasing evidence indicates that cervical failure in CI is not a single structural event but a biologically mediated process involving inflammatory activation, collagen disorganization, and altered extracellular matrix turnover. These historical technical adaptations can be interpreted as early clinical responses to biologic vulnerability of the cervix, in which hormonal, inflammatory, and extracellular matrix remodeling processes render the tissue progressively less capable of maintaining structural integrity.

At the tissue level, this vulnerability reflects dynamic remodeling of the cervical ex-tracellular matrix, characterized by progressive disorganization of collagen fibers, in-creased water content, and altered expression of matrix metalloproteinases. In parallel, immune activation and subclinical inflammation contribute to stromal softening and impaired tissue resilience. Hormonal signaling, particularly through progesterone withdrawal or functional resistance pathways, further modulates cervical compliance and barrier function. Collectively, these biologic processes precede and amplify biomechanical weakening, rendering the cervix increasingly susceptible to dilation under physiologic gestational load.

Earlier mechanical interpretations of CI tended to separate it from the broader pathways of preterm birth, limiting attention to the inflammatory and immunologic processes that contribute to cervical remodeling. Over the past two decades, however, growing evidence has shifted understanding toward a more integrated biomechanical–immunologic model in which tissue softening, extracellular-matrix reorganization, immune activation, and microbial or sterile inflammation interact to influence cervical competence. Within this framework, infection is no longer viewed strictly as a prohibitive state but rather as one biologic variable within a continuum of remodeling processes. This perspective does not propose a new disease mechanism; instead, it reflects an expanded interpretation of CI as part of a dynamic and multifactorial biologic pathway rather than a discrete structural defect.

Although suppression of microbial or inflammatory activity is essential for procedural safety, biologic stabilization alone does not reverse the cervical dilation or membrane prolapse already present. In such settings, mechanical reinforcement may still be required when structural indications persist, with infection control serving primarily to create safer conditions for intervention rather than altering the underlying anatomy. Observational studies support this distinction: Lee et al. [16] reported that delayed cerclage following infection control prolonged pregnancy by approximately eight weeks. More recently, Werlang et al. [17] emphasized the importance of infection-aware patient selection prior to rescue cerclage, demonstrating that amniotic fluid screening can identify subclinical intra-amniotic infection or inflammation and thereby refine candidacy for cerclage rather than attributing outcomes to cerclage alone. These findings suggest that targeted microbial suppression may enable safer application of mechanical support in selected patients, though they do not establish a specific therapeutic sequence or definitive causal effect. Diagnostic limitations also remain; current tools cannot reliably distinguish sterile inflammation from true intra-amniotic infection, and evidence supporting antibiotic regimens varies widely across studies. Consequently, decisions regarding timing and intervention must be individualized, integrating clinical presentation, cervical anatomy, and biologic context.

## 3. Historical and Contemporary Perspectives on Reinforcement Strategies

The conceptual approach to CI across major international guidelines has gradually broadened as understanding of cervical remodeling has advanced [18,19,20,21]. The RCOG guideline [18] describes the cervix as a functional barrier that contributes both mechanical closure and immunologic protection, reflecting a more integrated interpretation of cervical competence. Other guidelines, including ACOG [20] and NICE [21], continue to emphasize structural features in defining CI, illustrating the coexistence of complementary perspectives rather than a single uniform framework. A comparative summary of these guideline perspectives is provided in Table 1.

Table 1 summarizes these conceptual differences, outlining how major organizations including RCOG, SMFM, ACOG, and NICE describe cervical insufficiency within their respective frameworks. The guidelines vary in the extent to which CI is characterized as a discrete structural condition or considered within a broader continuum of factors contributing to preterm birth. Although none explicitly redefine CI as part of a dynamic biologic process, several incorporate elements that acknowledge functional competence, inflammatory modulation, and clinical context. These distinctions reflect complementary emphases rather than contradictory models, illustrating how differing clinical priorities shape the interpretation of cervical remodeling.

Among these guidelines, the RCOG [18] places relatively greater emphasis on the cervix as a functional barrier that incorporates both mechanical and immunologic elements, reflecting a broader interpretation of cervical competence. This perspective does not directly address specific reinforcement techniques but highlights components that conceptually align with contemporary views of cervical remodeling as a biologically modulated process. These elements complement rather than replace the structural focus found in other guidelines, illustrating the range of clinical lenses through which cervical insufficiency is understood.

**Figure 1:**
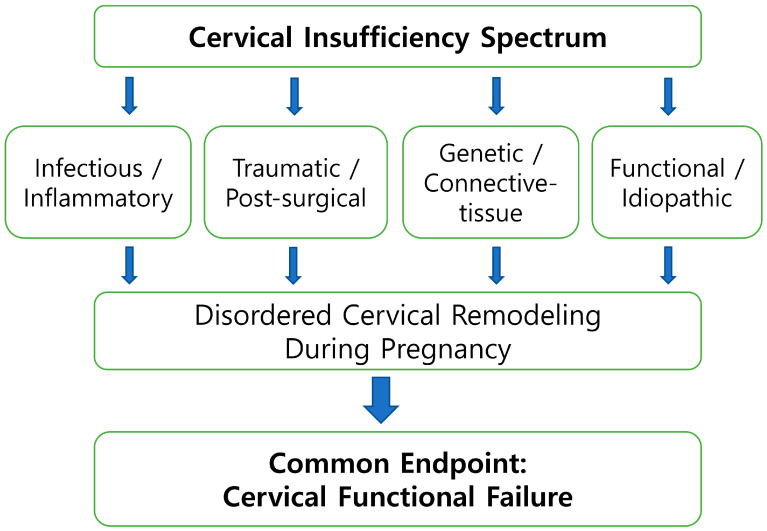
Etiologic heterogeneity of cervical insufficiency. Cervical insufficiency arises from multiple primary etiologic pathways, including infectious–inflammatory activation, traumatic or post-surgical disruption, genetic or connective-tissue vulnerability, and functional or idiopathic mechanisms. These distinct upstream pathways may converge on disordered cervical remodeling during pregnancy and ultimately lead to cervical functional failure. This figure provides a conceptual overview of etiologic diversity and convergence rather than a formal classification system.

To enhance clarity, Figure 1 provides a schematic illustration of how cervical remodeling can be interpreted within a dynamic continuum incorporating mechanical, inflammatory, and immunologic influences. This visual summary is intended to support conceptual understanding rather than to propose a new mechanistic model, and it complements the framework discussed in the accompanying text.

Figure 1 illustrates a schematic overview of cervical remodeling, depicting cervical insufficiency as a dynamic condition that may arise through multiple biologic and mechanical pathways within the preterm birth continuum. Rather than representing a single linear sequence, the figure highlights how diverse etiologic processes—including infectious–inflammatory activation, traumatic or post-surgical change, genetically mediated connective-tissue vulnerability, and functional or idiopathic factors—converge on disordered cervical remodeling during pregnancy, ultimately leading to cervical functional failure. This conceptual representation is intended to support a biologically integrative understanding of cervical change and does not imply a uniform mechanistic pathway. The framework is most applicable to inflammatory- and remodeling-driven presentations, and its relevance may be more limited in cases predominantly shaped by traumatic or genetic factors, which may follow distinct mechanobiologic trajectories.

**Figure 2:**
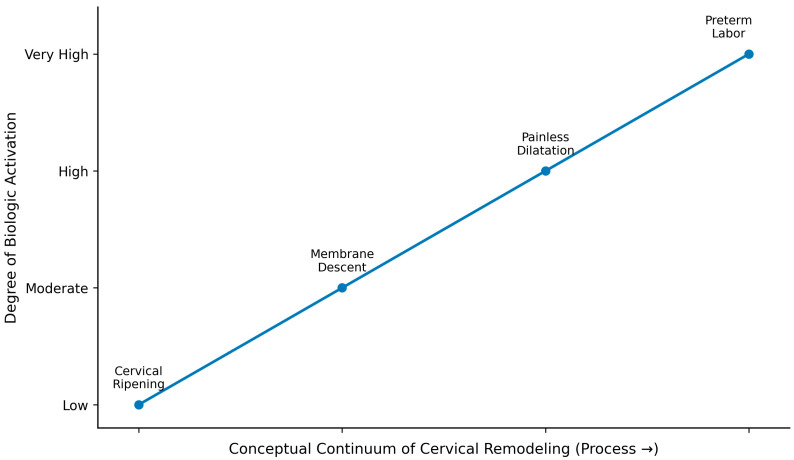
Conceptual continuum of cervical remodeling. This figure illustrates a conceptual continuum of cervical remodeling, reflecting increasing biologic activation from early tissue softening to inflammatory processes associated with preterm labor. The elements shown (cervical ripening, membrane descent, painless dilatation, and preterm labor) indicate mechanistic transitions rather than clinical stages. The continuum highlights progressive biologic activation that may arise across heterogeneous etiologies of cervical insufficiency, without implying a uniform sequence or inevitable progression.

Building on this etiologic framework, Figure 2 presents a conceptual continuum of cervical remodeling, illustrating progressive biologic activation from early softening to terminal inflammatory stages associated with preterm labor.

**Figure 3:**
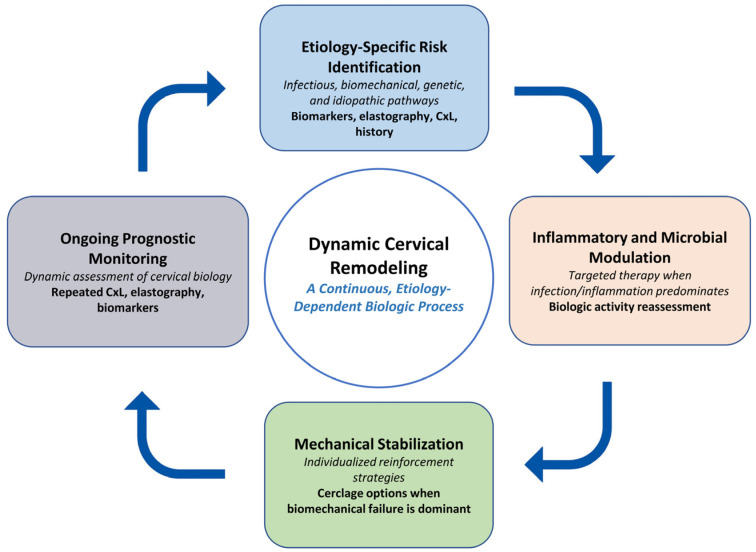
Conceptual feedback cycle of cervical remodeling. Cervical remodeling is illustrated as a dynamic, etiology-dependent process involving four interacting interpretive domains: (1) risk identification across infectious, biomechanical, genetic, and idiopathic pathways; (2) assessment of inflammatory or microbial activity when biologic activation predominates; (3) consideration of mechanical stabilization in contexts where biomechanical compromise is evident; and (4) ongoing prognostic monitoring through repeated evaluation of cervical length, elastography, and relevant biomarkers. This figure represents a conceptual framework intended to illustrate potential biologic feedback interactions rather than a management algorithm. Abbreviations: CxL, cervical length.

Table 2 provides a structured overview of how reinforcement strategies align with clinical context and available evidence. As indicated in the final row of Table 2, the integration of infection control, cerclage placement, and subsequent monitoring is presented as a conceptual management model rather than a procedural algorithm. Although not outcome-validated, this model reflects recurrent clinical patterns observed in women who exhibit dynamic cervical change rather than a single, static failure. This conceptual transition provides the foundation for Section 4, where we describe the proposed dynamic and cyclic management pathway in greater detail and present its visual representation in Figure 3.

## 4. Diagnostic and Prognostic Implications

A dynamic model of CI demands an equally dynamic diagnostic framework. Rather than relying solely on cervical length (CxL) or the presence of funneling, clinicians must interpret cervical changes in conjunction with biological and immunologic markers that reflect the ongoing remodeling process.

### 4.1. Diagnostic Biomarkers and Imaging of Cervical Remodeling

Modern obstetric diagnostics increasingly allow biologically informed assessment of cervical remodeling that extends beyond simple anatomic measurement. Cervical elastography, including shear-wave modalities, provides quantitative evaluation of cervical stiffness and collagen organization, offering insight into early biomechanical changes that may precede measurable cervical shortening [22,23]. Inflammatory biomarkers such as IL-6, MMP-8, and neutrophil elastase detected in cervical or vaginal secretions can reflect subclinical inflammatory activation and potential disruption of the mucosal barrier [24]. Amniotic fluid microbial testing, including qPCR and next-generation sequencing, contributes to distinguishing sterile inflammation from infection-driven remodeling, thereby informing whether infection-controlled cerclage may be safely considered in selected patients [25]. Collectively, these tools emphasize precision and biologic context, supporting earlier recognition of transitional remodeling phases rather than reliance solely on structural failure.

### 4.2. Diagnostic Workflow and Context-Specific Evaluation

Diagnostic interpretation in cervical insufficiency (CI) is strengthened by integrating biologic and biochemical context with structural assessment, rather than relying on cervical length as a solitary anatomic marker. In early or equivocal presentations, subtle cervical softening accompanied by supportive biomarker changes may suggest biologic activation and justify closer surveillance, with consideration of antimicrobial or anti-inflammatory measures when clinically indicated. In transitional presentations—often characterized by mid-trimester cervical shortening (e.g., cervical length ≤ 25 mm) or the presence of funneling on transvaginal ultrasound—the combined use of imaging with supportive biomarker data may provide a more informative assessment of ongoing cervical remodeling than cervical length alone [19]. In emergent presentations where amniotic membranes are exposed, pending culture results and in the absence of overt infection, clinical decision-making may consider the patient’s overall inflammatory status and trends in inflammatory markers such as C-reactive protein, while recognizing that RCOG does not mandate a specific biomarker-guided protocol for cerclage insertion [18]. Consistent with guideline-based recommendations—including NICE guidance recommending emergency cervical cerclage between 16 + 0 and 27 + 6 weeks in the presence of cervical dilation with exposed intact membranes—timely mechanical reinforcement may be considered, accompanied by context-appropriate consideration of antimicrobial management and multidisciplinary senior-level consultation [18,21]. When available, involvement of the most experienced clinician and, where appropriate, discussion with both obstetric and neonatal consultants should be undertaken. Referral to a specialist center with appropriate obstetric and neonatal expertise may be considered when local expertise or neonatal support is limited, to support complex decision-making. Subsequent reassessment should focus primarily on clinical status and obstetric evolution as the biologic context changes.

### 4.3. Prognostic Modeling

Emerging evidence suggests that diagnostic parameters, when interpreted longitudinally, may provide useful context for anticipating pregnancy course and refining the timing of intervention. Observational data indicate that trends in cervical length following infection control may be more informative than single measurements. Measures of residual cervical stiffness on elastography have been associated with lower risk signals for early delivery in selected cohorts, although validation is limited. Similarly, improvements in inflammatory profiles have been explored as potential indicators of clinical stabilization. These elements remain preliminary, but they illustrate how longitudinal biologic and biomechanical data could inform future prognostic frameworks. Conceptual models integrating these variables into composite assessments will require rigorous prospective validation before any clinical application can be considered.

### 4.4. Conceptual Implications for Clinical Decision-Making

Integrating emerging diagnostic and biologic insights into clinical interpretation may influence how CI is conceptualized in practice. Rather than relying solely on a binary designation of CI as “present” or “absent,” clinicians may increasingly view cervical change within a broader biologic context that reflects varying degrees of biomechanical and inflammatory activity. This perspective does not replace existing criteria but highlights how biologic information can complement structural assessment and support more individualized clinical reasoning.

In selected situations where cervical shortening or membrane descent recurs after an initial repair, individualized reassessment may support discussion of potential repeat intervention once infection control and anatomic feasibility have been confirmed. Prior reports have primarily described single-suture repeat procedures [26,27]. Such observations should be interpreted as exploratory and hypothesis-generating and do not constitute evidence of comparative effectiveness or procedural advantage.

The conceptual workflow depicted in Figure 3 illustrates how diagnostic findings, biologic indicators, and clinical assessment may be integrated in an iterative manner during the management of cervical insufficiency. Rather than outlining a prescriptive sequence, the figure emphasizes how evolving information regarding infection or inflammatory status, cervical imaging, and anatomic findings can inform subsequent clinical considerations. In circumstances where cervical shortening or membrane descent recurs after an initial intervention, reassessment of overall biologic context and anatomic feasibility may support individualized discussion of management options. This framework highlights that cervical change is often a dynamic process and may benefit from repeated evaluation rather than reliance on a single decision point. Although emergency cerclage is shown as an illustrative example, the broader principle of context-specific biologic reassessment may be conceptually applicable across diverse clinical scenarios. These concepts are derived primarily from observational evidence and are therefore presented as hypothesis-generating, pending further validation in prospective studies.

## 5. Conclusions and Future Directions

Cervical insufficiency (CI) is most coherently interpreted as a dynamic process situated within the continuum of preterm birth rather than as a fixed structural defect. Viewing cervical change through an integrated biomechanical–biologic lens allows structural compromise, inflammatory activation, and remodeling processes to be considered jointly, without displacing established diagnostic criteria.

From this perspective, CI may be understood as a condition that often evolves over time and, in selected contexts, retains the potential for stabilization when mechanical and biologic factors are addressed concurrently. Observations from contemporary multi-suture approaches illustrate one line of surgical inquiry within this biologic framework; however, the current evidence remains limited and predominantly observational. Clinically, this perspective encourages earlier attention to biologic context alongside anatomic findings and supports a more individualized approach to surveillance and intervention, particularly in patients with evolving or ambiguous disease presentations.

Future research would therefore benefit from prospective, multicenter investigations evaluating different cerclage approaches, including single- and double-suture techniques, within clearly defined diagnostic and infection-evaluation frameworks. Notably, the ongoing COSA trial (Cervical Occlusion double-level Stitch Application), a multicentre randomized study comparing double-level versus single-level emergency cerclage, represents a concrete effort to prospectively test whether reinforcement strategies translate into improved clinical outcomes [28]. Incorporation of cervical elastography, inflammatory biomarkers, and evaluation of mucosal barrier function may further clarify biologic transitions during cervical remodeling and help identify clinical contexts in which cervical change may remain potentially reversible.

Taken together, the perspectives advanced in this article support reframing CI as a modifiable, context-dependent condition embedded within a dynamic remodeling continuum. As the evidence base evolves, biologically informed approaches may contribute to a more coherent understanding of cervical remodeling across diverse clinical presentations and help refine future research priorities.

Despite broad recognition that cervical insufficiency represents a heterogeneous and biologically active process, translation of this understanding into guideline language has remained cautious. Several factors may contribute to this inertia, including the phenotypic heterogeneity encompassed by the term “cervical insufficiency,” the reliance of major guidelines on high-level interventional evidence rather than diagnostic or mechanistic frameworks, and the limitations of single-parameter assessment—such as cervical length alone—to capture inflammatory, infectious, and extracellular-matrix-related remodeling dynamics. Implementation considerations, including variability in clinical expertise and the need to balance timely intervention against potential overtreatment, may further constrain adoption of more process-oriented diagnostic formulations.

A shift in guideline perspective may therefore depend on evidence that is not only interventional but also diagnostically operationalizable. Prospective studies that stratify patients by biologically and mechanically defined phenotypes, integrate imaging-based measures with inflammatory or infectious biomarkers, and evaluate outcomes within clearly specified diagnostic pathways may help bridge the gap between conceptual advances and guideline formulation. In this context, the COSA trial (Cervical Occlusion Double-Level Stitch Application), a multicentre randomized study comparing double-level versus single-level emergency cerclage, represents an important step toward operationalizing level-based intervention strategies within a clearer diagnostic framework. The trial has recently been completed, although the results have not yet been published. By explicitly interrogating whether the configuration and level of mechanical reinforcement, rather than the decision to perform cerclage alone, influence pregnancy prolongation and perinatal outcomes, the study is directly aligned with the present framework. Once available, its findings may help clarify how diagnostic framing and procedural geometry interact within the preterm birth continuum and may inform future research design and guideline interpretation.

## Figures and Tables

**Table 1:** Conceptual Framing and Clinical Orientation of Major Guidelines on Cervical Insufficiency.

Organization/Guideline	Latest Version	Conceptual Framing of CI	Clinical Focus	Interpretation
RCOG Green-top Guideline No. 75 [18]	2023	Recognizes the cervix and cervical mucus plug as contributors to both mechanical integrity and host defense against ascending infection	Individualized cerclage indications; emphasis on infection assessment and control	Biologic and infection-aware conceptual framing
SMFM Consult Series #70 [19]	2024	Frames CI primarily in relation to cervical shortening, with limited acknowledgment of associated inflammatory processes	Focuses on management of asymptomatic short cervix, including cerclage decision-making	Predominantly phenotype-based framework, with limited integration of biologic considerations
ACOG Practice Bulletin No. 142 [20]	2014; reaffirmed 2023	Mechanical framework of the cervix	Traditional history-, ultrasound-, and exam-based indications	Predominantly mechanical framework maintained
NICE NG25 [21]	2019/updated 2022	Pragmatic coexistence of CI and short-cervix terminology	Recommends emergency cervical cerclage between 16 + 0 and 27 + 6 weeks with cervical dilation and exposed intact membranes	Predominantly stage-based clinical framework

Abbreviations: CI = cervical insufficiency.

**Table 2:** Evidence-supported vs. hypothesis-generating components within the dynamic cervical insufficiency framework.

Concept/Component	Evidence Level *	Evidence Type	Interpretation †
Infection-controlled cerclage	Moderate	Prospective cohorts; favorable trends after infection control	Evidence-supported
Delayed cerclage after infection control	Moderate	Prospective cohorts; similar findings across studies	Evidence-supported
Transvaginal cerclage (single-level)	Moderate	Observational cohorts across various clinical settings	Evidence-supported
Double-level cerclage (double McDonald, two-stitch techniques, and hybrid approaches)	Low–Moderate	Observational cohorts, mostly in ECC contexts; findings suggest possible benefit but techniques are heterogeneous	Evidence-supported with clinical consistency, but limited by study design
Transabdominal cerclage	Low–Moderate	Retrospective comparative studies with limited high-level evidence	Evidence-supported in selected populations, but limited by study design
Mucus-plug reinforcement/preservation mechanism	Low	Mechanistic plausibility; limited direct evidence	Hypothesis-generating
Cervical elastography for early biomechanical weakening	Moderate	Meta-analyses; consistent diagnostic accuracy	Evidence-supported
Biomarkers (IL-6, MMP-8) as indicators of subclinical inflammation	Moderate	Observational studies with similar associations	Evidence-supported
Cervical remodeling as a biomechanical–immunologic continuum	Conceptual	Integrative interpretation of existing biological data	Hypothesis-generating
Dynamic, cyclic management model integrating infection control → cerclage → monitoring	Conceptual	Systems-level conceptual model without outcome-level validation	Hypothesis-generating

* Evidence level categories adapted from simplified GRADE descriptors: Moderate/Low–Moderate/Low/Conceptual. Abbreviations: ECC, emergency cervical cerclage. † Interpretation statements in this table reflect the authors’ synthesis of the available literature and do not represent formal evidence grading systems or official statements from clinical guidelines.

## Data Availability

No new data were created or analyzed in this study. Data sharing is not applicable to this article.

## References

[1] Romero R., Espinoza J., Erez O., Hassan S.S. (2006). The role of cervical cerclage in obstetric practice: Can the patient who could benefit from this procedure be identified ?. Am. J. Obstet. Gynecol..

[2] Lee S.E., Romero R., Park C.W., Jun J.K., Yoon B.H. (2008). The frequency and significance of intraamniotic inflammation in patients with cervical insufficiency. Am. J. Obstet. Gynecol..

[3] Vink J., Feltovich H. (2016). Cervical etiology of spontaneous preterm birth. Semin. Fetal Neonatal Med..

[4] Tantengco O.A.G., Menon R. (2020). Contractile function of the cervix plays a role in normal and pathological pregnancy and parturition. Med. Hypotheses.

[5] Casarini L., Santi D., Marino M. (2015). Impact of gene polymorphisms of gonadotropins and their receptors on human reproductive success. Reproduction.

[6] Amabebe E., Ogidi H., Anumba D.O. (2022). Matrix metalloproteinase-induced cervical extracellular matrix remodelling in pregnancy and cervical cancer. Reprod. Fertil..

[7] Gomez-Lopez N., Galaz J., Miller D., Farias-Jofre M., Liu Z., Arenas-Hernandez M., Garcia-Flores V., Shaffer Z., Greenberg J.M., Theis K.R. (2022). The immunobiology of preterm labor and birth: Intra-amniotic inflammation or breakdown of maternal-fetal homeostasis. Reproduction.

[8] Hochuli E., Vogt H.P. (1987). Die “Doppelcerclage”. Die Zervixinsuffizienz im mittleren Trimester und ihre Behandlung [Double cerclage. Cervix insufficiency in the middle trimester and its treatment]. Geburtshilfe Frauenheilkd.

[9] Park M.I., Lee M.H., Kong M.S., Hwang J.H., Chung S.R., Moon H. (2000). Clinical experience of 15 cases of modified McDonald cerclage using Beriplast™ in incompetent internal os of cervix. Korean J. Obstet. Gynecol..

[10] Vaughan M., Emms A., Hodgetts Morton V., Morris R.K., Israfil-Bayli F., Pilarski N. (2025). Optimal surgical technique at cervical cerclage to prevent pregnancy loss, a systematic review. Eur. J. Obstet. Gynecol. Reprod. Biol..

[11] Donadono V., Koutikwar P., Banerjee A., Ivan M., Colley C.S., Sciacca M., Casagrandi D., Tetteh A., Greenwold N., Kindinger L.M. (2025). Transvaginal cervical cerclage: Double monofilament modified Wurm vs single braided McDonald technique. Ultrasound Obstet. Gynecol..

[12] Xu Z.M., Zhang J., Hong X.L., Liu J., Yang Z., Pan M. (2023). Comparison of two stitches versus one stitch for emergency cervical cerclage to prevent preterm birth in singleton pregnancies. Int. J. Gynaecol. Obstet..

[13] Qiu L., Lv M., Chen L., Chen Z., Shen J., Wang M., Cai Y., Zhao B., Luo Q. (2024). Comparison of two emergency cervical cerclage techniques in twin pregnancies: A retrospective cohort study matched with cervical dilation. Int. J. Gynaecol. Obstet..

[14] Park Y.J., Park M.I. (2025). Double versus single cervical cerclage in women with cervical insufficiency: A systematic review of prophylactic and emergency indications. Reprod. Med..

[15] Temming L.A., Mikhail E., Society for Maternal-Fetal Medicine (SMFM) Publications Committee (2023). Society for Maternal-Fetal Medicine Consult Series #65: Transabdominal cerclage. Am. J. Obstet. Gynecol..

[16] Lee K.N., Park K.H., Kim Y.M., Cho I., Kim T.E. (2022). Prediction of emergency cerclage outcomes in women with cervical insufficiency: The role of inflammatory, angiogenic, and extracellular matrix-related proteins in amniotic fluid. PLoS ONE.

[17] Werlang A., De Simone A., Jones G. (2025). Amniocentesis and therapeutic amnioreduction before rescue cerclage: Improving patient selection for rescue cerclage based on amniotic fluid screening. J. Obstet. Gynaecol. Can..

[18] Royal College of Obstetricians and Gynaecologists (RCOG) (2023). Cervical Cerclage (Green-Top Guideline No.75).

[19] Society for Maternal-Fetal Medicine (SMFM) (2024). SMFM Consult Series #70: Management of short cervix in individuals without a history of spontaneous preterm birth. Am J Obstet Gynecol..

[20] American College of Obstetricians and Gynecologists (ACOG) (2014). Cerclage for the management of cervical insufficiency (Practice Bulletin No.142). Obstet. Gynecol..

[21] National Institute for Health and Care Excellence (NICE) (2019). Preterm Labour and Birth (NG25).

[22] Wang B., Zhang Y., Chen S., Xiang X., Wen J., Yi M., He B., Hu B. (2019). Diagnostic accuracy of cervical elastography in predicting preterm delivery: A systematic review and meta-analysis. Medicine.

[23] Angelopoulou E., Gourounti K., Bolou A., Manesi M., Diamanti A. (2025). Cervical elastography as a predictive tool for preterm birth: A systematic review and meta-analysis. Cureus.

[24] Romero R., Espinoza J., Gonçalves L.F., Kusanovic J.P., Friel L.A., Nien J.K. (2006). Inflammation in preterm and term labour and delivery. Semin. Fetal Neonatal Med..

[25] DiGiulio D.B., Romero R., Amogan H.P., Kusanovic J.P., Bik E.M., Gotsch F., Kim C.J., Erez O., Edwin S., Relman D.A. (2008). Microbial prevalence, diversity and abundance in amniotic fluid during preterm labor: A molecular and culture-based investigation. PLoS ONE.

[26] Song J.-E., Lee K.-Y., Jun H.-A. (2011). Repeat cerclage prolongs pregnancy in women with prolapsed membranes after prior cerclage. Acta Obstet. Gynecol. Scand..

[27] Ru P., Ni X., Xu W., Liu Y., Meng L., Yuan W., Gu Z., Shi J., Su X., Liu M. (2024). Perinatal outcomes in patients undergoing repeat cerclage: A retrospective case series. Int. J. Gynaecol. Obstet..

[28] Kosińska-Kaczyńska K., Rebizant B., Bednarek K., Dabrowski F., Kajdy A., Muzyka-Placzyńska K., Filipecka-Tyczka D., Uzar P., Kwiatkowski S., Torbe A. (2023). Emergency cerclage using double-level versus single-level suture in the management of cervical insufficiency (Cervical Occlusion double-level Stitch Application, COSA): Study protocol for a multicentre, non-blinded, randomised controlled trial. BMJ Open.

